# The Role of Anabolic Androgenic Steroids in Disruption of the Physiological Function in Discrete Areas of the Central Nervous System

**DOI:** 10.1007/s12035-017-0774-1

**Published:** 2017-10-02

**Authors:** Giuseppe Bertozzi, Francesco Sessa, Giuseppe Davide Albano, Gabriele Sani, Francesca Maglietta, Mohsin H. K. Roshan, Giovanni Li Volti, Renato Bernardini, Roberto Avola, Cristoforo Pomara, Monica Salerno

**Affiliations:** 1 Department of Clinical and Experimental Medicine, Section of Forensic Pathology, University of Foggia, Ospedale Colonnello D’Avanzo, Foggia, Italy; 2 NESMOS Department (Neurosciences, Mental Health, and Sensory Organs), Sapienza University of Rome, School of Medicine and Psychology, Sant’Andrea Hospital, Rome, Italy; 3 Centro Lucio Bini, Rome, Italy; 4 Department of Anatomy, School of Medicine, University of Malta, Msida, Malta; 5 Department of Biomedical and Biotechnological Sciences, University of Catania, Catania, Italy; 6 D’Avanzo Hospital, 71122 Foggia, Italy

**Keywords:** Anabolic-androgenic steroids (AAS), Abuse, Molecular mechanisms, Behavioral disorders, Amygdala, Central nervous system

## Abstract

Anabolic-androgenic steroids (AAS) abuse is often associated with a wide spectrum of adverse effects. These drugs are frequently abused by adolescents and athletes for esthetic purposes, as well as for improvement of their endurance and performances. In this literature review, we evaluated the correlation between AAS and anxiety or aggression. Two pathways are thought to be involved in AAS-induced behavioral disorders. Direct pathway via the amygdalo-fugal pathway, which connects the central nucleus of the amygdala to the brainstem, is involved in cognitive-emotive and homeostatic processes. The latter is modified by chronic AAS use, which subsequently leads to increased anxiety. Indirect pathways via the serotonergic, dopaminergic, and glutamatergic signals which are modified by AAS abuse in latero-anterior hypothalamus and can mediate the aggressive behavior. In conclusion, the molecular mechanisms underlying the behavioral alterations following AAS abuse is unclear and remains ambiguous as additional long-term studies aimed to understand the precise mechanisms are required.

## Introduction

**Fig. 1:**
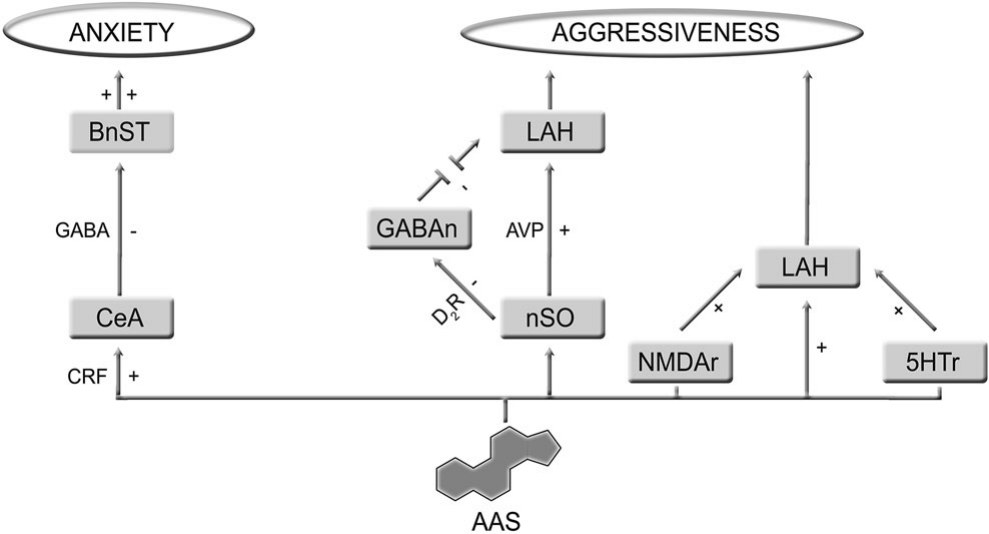
Cerebral structure involved in AAS-induced damage and their connections. The pathway thought to determine AAS-induced behavioral disorders concerned the amygdalo-fugal signaling, which connects the central amygdala (CeA) to bed nucleus of the stria terminalis (BnST) involves supraoptic neurons (nSO) and affects latero-anterior hypothalamus (LAH)

Anabolic-androgenic steroids (AAS) are testosterone-derived molecules which are often drug of abuse, and studies have shown their relation to a broad spectrum of side effects, including behavioral disorders [1]. With regard to this, at low concentrations, AAS has trivial effects on the mood, as well as perceived to be clinically beneficial when used in accordance with the treatment protocols for dysthymia and refractory depression. Nonetheless, at supraphysiological doses, AAS consumption is associated with depression, hypomania, or mania [2, 3]. AAS-mediated behavior disorders may simply reflect the notable psychopathological comorbidity between drug addiction and disorders inducing neuroadaptive and neurotrophic changes in neural circuits [4]. Piacentino et al. suggested that the relationship between AAS use and psychopathology cannot be considered as a mere linear relationship, instead, it is considered to be more than a complex circuitry, driven by neuroendocrine mechanism, which involves different areas of the central nervous system (CNS) in addition to the involvement of the hypothalamic-pituitary-adrenal axis (HPA) [5]. AAS exert their pharmacological effects on the CNS in two distinctive ways: directly, by modulating their own intracellular receptors; and indirectly, by either influencing the binding site located on the neurotransmitter receptor or by causing the release of neuropeptides [6, 7]. These mechanisms are, thereby, affected by the expression of inhibitory GABA receptors and 5-HT receptors, which, among others, are abundantly expressed in areas of the brain associated with depression, stress, anger, and sexual behavior [8, 9]. A number of studies have shown a significant interaction between 5-HTT-linked polymorphic region (5-HTTLPR) and neuroticism in athletes predisposed to anxiety and depression [10]. In addition, peripheral circulating AAS may be converted into estrogen derivatives, therefore, activating second messenger systems. Most AAS binds to androgen receptors and can also be aromatized to estrogenic metabolites which are, in turn, able to interact with both estrogen receptor-alpha (ERα) and estrogen receptor-beta (ERβ). Furthermore, AAS can also signal via the ERα, ERβ, and progesterone receptors, without being metabolically converted to estrogen [11]. Areas of the brain involved in the onset of anxiety and aggression associated with AAS abuse appear to have an affluent expression of steroid receptors and of enzymes involved in steroid synthesis and metabolism [12, 13]. Cerebral structures involved in AAS-induced damage include the hypothalamus, basal ganglia, the amygdala, and the hippocampus. They allow the configuration of the underlying neurotransmitter/neuropeptide-driven integration network, which substantially contributes to anxiety- and aggressive-like behavior in animal model (Fig. 1). In details, the extended amygdala seems to play a major role in such mechanisms, and questions have been raised on its role in anxiety syndrome progression, particularly the involvement of subdivisions of the extended amygdala which includes the central amygdala (CeA) and bed nucleus of the stria terminalis (BnST). Furthermore, an imbalance in such relationship, mainly those involving the corticotropin-releasing hormone (CRH) transcripts and GABAergic nerve endings, may mediate anxiogenic behavior in mice [14]. Moreover, the lateral hypothalamic area is activated during aggression following AAS administration and abuse [15, 16]. Lateral hypothalamic is in fact regarded as the converging point where different neurotransmitters and their receptor converge to exert their effects. The result of such convergence between excitatory and inhibitory stimuli occurring at this level makes the lateral hypothalamus an ideal area to study the biological mechanisms underlying the induction of aggression occurring in AAS abusers.

Indeed, similar behavioral alterations in AAS abusers have been found and confirmed by the Center for Disease Control and Prevention (CDC), which reported that approximately two million individuals currently use or have used AAS in the USA, suggesting the AAS consumption is rapidly becoming pandemic worldwide [17]. Furthermore, *Pope and Katz* interviewed around 41 bodybuilders and football players, who were identified as AAS abusers. The prevalence of mood disorders was found in 22% of these individuals [18, 19]. Other findings reported by CDC included 23% of athletes who were AAS abusers and met the Diagnostic and Statistical Manual of Mental Disorder III (DSM-III) criteria for mood disorders, including major depressive and bipolar disorders. In addition, such relationship was found to be significantly higher in athletes who took AAS regularly and at supraphysiological doses, compared to naïve athletes, exhibiting a prevalence of only 4% for major mood disorders [18]. Thus, it is reasonable to hypothesize that the AAS-related psychopathological syndrome is dose-dependent. Data correlating with such hypothesis was reported in a controlled retrospective study by Perry et al., which compared 20 regular AAS consuming male weightlifters to 20 non-AAS consuming male weightlifters, and showed that the formers exhibited a more depressive profile than the latter [20, 21].

Moreover, symptoms were more prominent and clinically substantial in AAS users compared to non-AAS subjects. In addition, a survey conducted on a cohort of 479 athletes recruited via the Internet fitness forums, bodybuilding, weightlifting, and steroid websites showed that 16% of male AAS-dependent users had a history of DSM-IV-TR anxiety disorders, comprised of either generalized anxiety disorder (GAD), panic disorder, post-traumatic stress disorder (PTSD), obsessive-compulsive disorder (OCD), or social phobia [22]. Furthermore, in order to determine the age variability among the AAS consumers and abusers, Ip et al. recruited age-matched (40 or over) sportsmen and habitual steroid users, who admitted using AAS for non-fitness purposes. Results obtained from the study suggested that anxiety disorders were more prevalent among the AAS users, who were also engaged in aggressive alcohol use and polypharmacy compared to non-AAS users [23]. Likewise, Sato et al. described that AAS abuse in adolescence may alter the levels of androgens, which then predispose these individuals to an increased level of anxiety and altered response to stress [24].

AAS use has also been proven to unmask behavioral disorders, such as increased aggression, hostility, and unprovoked rage attacks. These emotional and behavioral outbursts have been referred to as “roid rage,” defined as sudden and exaggerated aggression induced by AAS use which is prone to a subliminal provocation [24, 25]. In addition, AAS users often suffer from a vigilant mindset accompanied by inability to adjust to situations, which then results in frustration and impulsiveness. Moreover, AAS use has also been associated to a wide range of violent crimes and domestic violence, particularly, physical abuse of the partner [5].

The aim of this literature review is to highlight the recent understanding and advances made regarding the mechanisms involved in AAS-induced disequilibrium in discrete areas of the brain, as well as to shed some light on the roles which neurotransmitters plays in the dysregulation process (Table 1), which then leads to substantial disruption of the physiological homeostasis and ultimately causes brain dysfunction and behavioral impairment.

**Table 1:** Previously animal studies on AAS administration and their neurobiological effects

Study	Population	AAS used	Findings
Schwartzer JJ et al. (2009) [26]	Male Syrian hamsters (Charles River Laboratories, Wilmington, MA).	Mixture of testosterone cypionate, nandrolone, boldenone (1-dehydrotestosterone) (Steraloids Inc., Newport, RI).	AAS produce an increase number of D2-ir and GAD67-ir cells and a decrease of GABAA-ir elements in the LAH.
Kindlundh et al. (2003) [9]	Male Sprague–Dawley rats (Alab, Sollentuna, Sweden).	Nandrolone decanoate (Deca-Durabol, Organon, Oss, the Netherlands).	AAS promote a significant downregulation of the 5HT1B receptor density in hippocampus and in the medial globus pallidus and a significant upregulation of the 5HT2 receptor density in the nucleus accumbens shell.
Melloni and Ricci (2010) [15]	Pubertal male Syrian hamsters (Charles River Laboratories, Wilmington, MA).	Mixture of testosterone cypionate, nortestosterone, and dihydrotestosterone undecyclate (Steraloids, Newport, RI).	AAS induce differences in aggressive responding between exposed and withdrawal periods, with a decrease in aggressive behavior and an increase in anxiety-like responding.
Oberlander and Henderson (2012) [11]	Female C57BL/6J mice (Jackson Laboratories, Bar Harbor, ME).	Mixture of testosterone cypionate (Sigma; St Louis, MO), nandrolone decanoate (Sigma), and methandrostenolone (Steraloids; Newport, RI).	Chronic AAS administration increases CRF mRNA concentration in the CeA and CRF-1R in the BnST, enhancing inhibitory CRF-dependent activity of GABAergic afferents from CeA to the BnST.
Costine et al. (2010) [27]	C57Bl/6 mice (Charles River Laboratory; Wilmington, MA, USA) or C57Bl/6J (Jackson Laboratories; Bar Harbor, ME, USA)	Mixture of testosterone cypionate (Sigma, St. Louis, MO, USA), 19-nortestosterone derivatives (nandrolone decanoate; Sigma) and methandrostenolone (Steraloids, Newport, RI, USA).	AAS increased the levels of CRF mRNA and CRF immunoreactivity in the CeA, as well as immunoreactivity in the dorsal aspect of the anterolateral division of the bed nucleus of the stria terminalis (BnST).
Ambar and Chiavegatto (2009) [28]	Male C57BL/6J mice (University of Sao Paulo Medical School, Brazil).	Nandrolone decanoate (Decadurabolin, Organon, Brazil).	AAS reduced mRNA levels of postsynaptic 5-HTreceptors in the amygdala and prefrontal cortex, with the 5-HT1B mRNA level more reduced in the hippocampus and hypothalamus.
Masonis and McCarthy (1995) [29]	Synaptoneurosomal membrane preparations from male and female, Sprague–Dawley rats (Hilltop Lab Animals Inc., Scottsdale, PA).	Stanozolol and 17a-methyltestosterone (17a-MT).	AAS directly interact with the GABA receptor.
Henderson (2007) [30]		Effects of 17α-MeT.	AAS enhance GABA activity via allosteric binding to α_2_-containing receptors.
Schwartzer et al. (2009) [31]	Male P21 hamsters (Charles River Laboratories, Wilmington, MA).	Mixture of testosterone cypionate, nortestosterone, and dihydrotestosterone undecylate (Steraloids Inc., Newport, RI).	AAS administration is related to significant increases in 5-HT2A fibers in the lateral portion of the anterior hypothalamus (LAH) with a similar significant increase in the number of cells expressing 5-HT2A-ir in the LAH.
Harrison et al. (2000) [32]	Adolescent male hamsters (P25) (Harlan Sprague–Dawley Labs, Indianapolis, IN).	Mixture of testosterone cypionate, nandrolone deconate, boldenone undecylenate (Sigma Chemical Co., St. Louis, MO).	AAS cause no difference in the distribution and number of AVP neurons, AVP fiber density, and peptide content; however, a darker staining for AVP in the mSON, and a more dense pattern of fiber staining for AVP in the AH brain region is revealed.
DeLeon et al. (2002) [33]	Adolescent male hamsters (P25) (Harlan Sprague–Dawley Labs, Indianapolis, IN).	Mixture of testosterone cypionate, nandrolone deconate, boldenone undecylenate (Sigma Chemical Co., St. Louis, MO).	Chronic AAS exposure can increase the binding of AVP V1A receptors in several areas of the hamster brain implicated in aggressive responding.
Carrillo et al. (2009) [34]	Male Syrian hamsters postnatal day 21 (P21) were obtained from Harlan Sprague–Dawley, Inc. (Indianapolis, IN).	Mixture of testosterone cypionate, nortestosterone, dihydrotestosterone undecyclate (Steraloids, Newport, RI).	AAS significantly increase in phosphate-activated glutaminase immunoreactivity (PAG-IR) and FOS/PAG-IR in the LAH, as well as decrease afferent innervation from the LAH to the VLH.
Fischer et al. (2007) [35]	Prepubertal male Syrian hamsters (P21–P23) (Charles River Laboratories, Wilmington, MA).	Mixture of testosterone cypionate, nortestosterone, dihydroxytestosterone undecylate (Steraloids Inc., Newport, R.I.).	AAS administration provokes a significant increase in the number of PAG- and GluR1-containing neurons in aggression brain areas.
Rossbach et al. (2007) [36]	Male Sprague–Dawley rats (Alab, Sollentuna, Sweden).	Nandrolone decanoate (Deca-Durabol Organon, Oss, the Netherlands).	AAS increase phosphorylation of the NMDA receptor subunits NR2A and NR2B and ERK1/2.
Le Grevès et al. (1997) [37]	Male Sprague–Dawley rats (Alab, Sweden).	Nandrolone decanoate (Deca-Durabol, Organon, Oss, the Netherlands).	AAS produce a significant mRNA expression decrease of the NR2A receptor subunit both in the hypothalamus and hippocampus. NR1 receptor is affected by higher dose of AAS in the nucleus accumbens.
Birgner et al. (2008) [38]		Effects of nandrolone decanoate.	AAS adminitrastion determine an expression increase of the dopamine D1-receptor in the amygdala and decrease in the hippocampus, while transcription levels of the dopamine D4-receptor was increased in the nucleus accumbens.
Kindlundh et al. (2003) [9]	Male Sprague–Dawley rats (Alab, Sollentuna, Sweden).	Nandrolone decanoate (Deca-Durabol, Organon, Oss, the Netherlands).	AAS influence mRNA levels of both the dopamine D1-receptor subtype (significantly reduced in the caudate putamen and at high doses in the nucleus accumbens shell) and the dopamine D2-receptor (significantly increased at low doses in the caudate putamen and the nucleus accumbens shell).
Schwartzer and Melloni (2010) [26]	Male Syrian hamsters (Charles River Laboratories, Wilmington, MA).	Mixture of testosterone cypionate, nandrolone decanoate, and boldenone undecylenate (Steraloids Inc., Newport, RI).	AAS treated brains show a colocalization of GAD_67_ neurons and D5 receptors in the LAH. In addition, local infusion of D2 antagonist (eticlopride) into the AH provoke a dose-dependent suppression of aggressive behavior.

## AAS and Cerebral Mechanisms on Anxiety

Neural circuits involved in AAS-induced GAD are referred to as the extended amygdale [14]. It constitutes as the latter part of the limbic system and is composed of the CeA, the shell of the nucleus accumbens, and the BnST. Moreover, CeA has been postulated to be related to specific threat phasic fear. On the other hand, BnST has been considered to play an important role in sustained anxiety, since the disequilibrium between these two anatomical structures may lead to anxiety. The extended amygdala integrates cognitive-emotive and homeostatic processes by releasing an array of diverse neuromodulators, such as CRH and brain-derived neurotrophic factor, as well as neurotransmitters, including GABA, glutamate, serotonin (5HT), and dopamine [27, 28, 39–41].

**Fig. 2:**
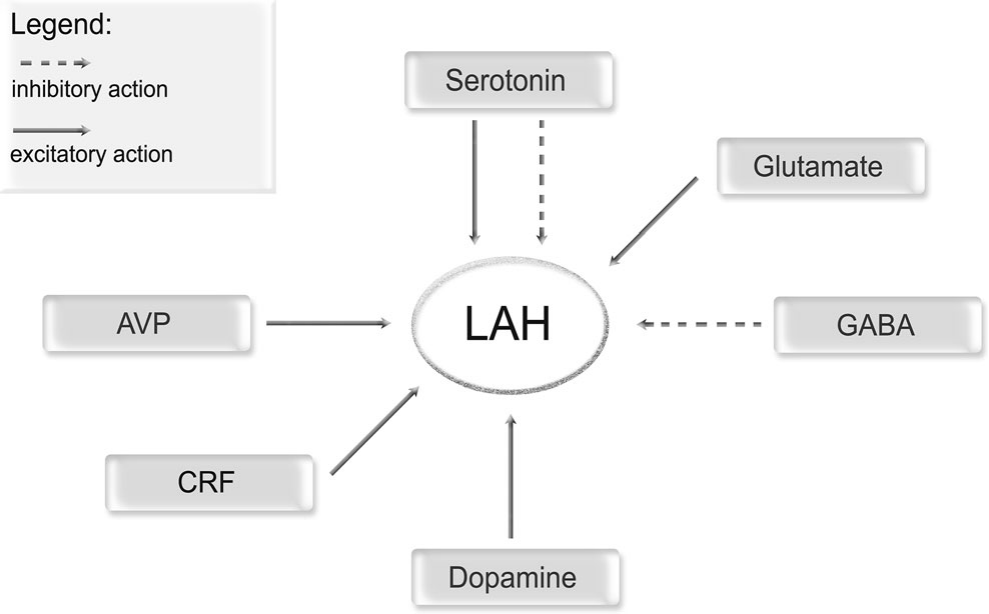
Chronic administration of high doses of anabolic-androgenic steroids (AAS**)** promote anxiety-like behavior, through corticotrophin release factor (CRF) by enhancing GABAergic inhibitory effects from central amygdala (CeA**)** onto bed nucleus of the stria terminalis (BnST). Moreover, chronic AAS administration alters neurotransmitter expression involved in aggression control. AAS enhanced D_2_R-mediated activation from supraoptic neurons (nSO) onto latero-anterior hypothalamus (LAH) and blocked GABA-mediated inhibition of AVP cells. In addition, AAS promote inverse relationship between 5-HT_1A_-R-induced inhibition and 5-HT_2A_-R-induced excitation, modifying their expression in hypothalamus. Finally, AAS are able to induce NMDA receptor phosphorylation in order to increase excitatory neurotransmission, resulting in an increment of aggression

Studies in male rat suggested that a prolonged exposure to AAS at supraphysiological doses may promote anxiety-like behavior, which is prevented or attenuated by intracerebroventricular injection of the CRH receptor type-1 antagonist (antalarmin) [9]. This process was characterized by an enhanced GABAergic activity in neurons of the CeA, which thereby, exerted an inhibitory effect on BnST, in addition to being also inhibited by intracerebroventricular infusion of either alarmin, or GABA_A_R-1 antagonist picrotoxin. In addition, BnST activity can be sustained by CRH administration. This finding advocates a possible relationship exists between GABA, CRH, and anxiety-like behavior, at least in these experimental animal models. Furthermore, increased CRH mRNA transcription was also detected in CeA neurons after chronic AAS administration [42]. Therefore, the sequence of events leading to AAS-induced anxiogenic behavior is as following: AAS enhance CRHR-1-mediated presynaptic GABA release from CeA to BnST; GABA modulates the activity of the CeA-BnST connection via an inhibitory pathway, and such disequilibrium then leads to anxiogenic behavior (Fig. 2) [43].

Nandrolone is one of the most common AAS used worldwide, possesses stimulatory properties for synaptic transmission, via GABA_A_ receptors in the ventromedial nucleus of the hypothalamus but causes GABA_A_ receptors to diminish in pre-optic areas. Furthermore, the increased release of neurons from CeA projecting to BnST and GABAergic-A receptor-mediated inhibition to BnST neurons has been associated to the development of aggression, stress, and anxiety [29, 44]. These effects are strictly related to the GABA_A_ receptor, a pentameric transmembrane protein encoded by 16 different receptor subunit genes (α1–α6, β1–β3, γ1–γ3, δ, ε, π, and θ) [29]. Among the possible combinations, α2 subunit-containing receptors seem to be highly expressed in the extended amygdala, where they appear to orchestrate the sequence of events that eventually leads the development of anxiety behavior. Indeed, an increase in GABAergic activity via the α2-containing receptors was evoked with the administration of 17α-methyltestosterone, suggesting a possible direct allosteric modulation of these GABAergic receptors in the extended amygdala, which results in acute anxiolytic activities [30]. Furthermore, blockade and enhancement of synaptic conduction in dorsomedial hypothalamus is associated subsequently with anxiogenic responses and anxiolysis [45, 46]. However, modification of the physiological GABA_A_-mediated neural signal is not easy nor does it occur so consistently, therefore, suggesting a key role of GABA_A_ receptors in provoking anxiety-like disorders. The complex relationship between AAS and specific areas of the brain, such as hypothalamus, basal ganglia, amygdala, hippocampus, brain stem, spinal cord, and cerebral cortex, may reflect the involvement and interactions of a multi-neurotransmitter system which includes the serotonergic, glutamatergic, and dopaminergic pathways, subsequently leading to anxiety and aggression.

## AAS and Cerebral Mechanism of Aggressiveness

Prolonged administration of AAS is associated with altered molecular expression of ERα or ERβ receptors in regions of the brain responsible for the control of aggression, and, among them, the important regions which lie within the latero-anterior hypothalamic area [15]. Furthermore, administration of AAS in animal model at given dose and time intervals reproduces the effects comparable to those seen in AAS abusers and induces changes in serotonergic, dopaminergic, and glutamatergic signaling, thus leading to anxiety-like behavior [4, 47].

The central serotonergic system has a pivotal role in the regulation of the activity of the central nervous system as well as influences a wide range of physiological and psychological processes, including the personality traits: for instance, low levels of 5-HT has been associated with impulsive aggression in both human and animal studies [48]. Therefore, chronic AAS use may lead to a decrease in 5-HT_1A_ receptor (5-HT_1A_-R) expression, as well as upregulation and increase activity of 5-HT_2A_-R, in the same regions of the latero-anterior hypothalamus [31, 49]. Such reciprocal relationship between inhibition of the 5-HT_1A_-R and upregulation of 5-HT_2A_-R represents nearly the major contribution of AAS to the pathological development of aggression in the hypothalamus. Furthermore, 5-HT_1A_-R is located in the hippocampus, septum, amygdala, hypothalamus, and neocortex. The limbic system encompasses most of the latter areas and in fact is involved in modulating the emotional behavior. In addition, the presence of 5-HT_1A_-R in the neocortex suggests that 5-HT_1A_-R likely contributes to cortical cognitive and/or integrative functions. 5-HT_1A_-R is also amply expressed in both the dorsal and the median raphe nuclei, where they act as autoreceptors and modulates signal transmission of the serotonergic neurons. The 5-HT_1B_-R is expressed exclusively in the rodent brain, whereas its analog 5-HT_1D_-R is found in both bovine and humans. They are typically distributed in the dorsal raphe nucleus at the level of presynaptic terminals of serotonergic neurons, and they are thought to modulate serotonin release. In postsynaptic neurons, both 5-HT_1B_-R and 5-HT_1D_-R modulates the release of other neurotransmitters, such as acetylcholine (ACh) in the hippocampus and dopamine (DA) in the prefrontal cortex. On the other hand, 5-HT_2A_ receptors are found in various cortical regions, particularly in the frontal cortex, as well as in discrete areas of the limbic system (Fig. 2).

Moreover, 5-HT neurons in the dorsal raphe nuclei have been hypothesized to interact with hypothalamic neurons to cause the release of vasopressin (AVP), in order to facilitate aggressiveness [15]. The cerebral regions primarily involved in aggressive behavior are located in the hypothalamus. Harrison et al. demonstrated that hamster exposed to AAS during adolescent is at risk of developing increased aggressive behavior, since AAS influences the anterior hypothalamic-arginine vasopressin (AH-AVP) neural circuitry. It does so by increasing the AH-AVP fiber density and peptide content, which subsequently leads to an increase aggression intensity. In light of this, we may conclude that an existing underlying relationship between AAS exposure and AH-AVP expression and activity is needed to stimulate early onset of AAS-stimulated aggression [32]. Furthermore, early exposure of AAS in adolescents may increase vasopressin (1A) receptor binding in several significant areas of the hamster brain which may facilitate the aggressive behavior [33].

In addition, AAS consumption during adolescences may modify the physiological activity of the glutamatergic system in latero-anterior hypothalamus (LAH), a structure involved in development of aggressive behavior [34, 35]. Rossbach et al. demonstrated that AAS induces NMDA receptor phosphorylation that leads to an increase in excitatory neurotransmission in rodents, thereby, resulting in an increase aggression, impulsiveness, and irritability in both adult and adolescent rats [36]. NMDA and sigma receptor agonists, dehydroepiandrosterone sulfate, pregnenolone sulfate, and pregnenolone sulfate used in other studies managed to displace the natural receptor agonists from both sigma-1 and sigma-2 sites. Chronic nandrolone administration, on the other hand, only managed to alter the affinity of the sigma-1 receptor, but not of the sigma-2 receptor [50].

NMDA receptor is a heterotetramer, consisting of different subunits, which are comprised of one GluN1 subunit, four GluN2 subunits (GluN2A, GluN2B, GluN2C, and GluN2D) encoded by four different genes. The two GluN3 subunits (GluN3A and GluN3B) are encoded by two different genes. Moreover, the NMDAR structure is typically formed by a GluN1 and GluN2 subunits or by a combination of both GluN2 and GluN3 subunits [51]. A recent study by Le Grevès et al. suggested that AAS administration induced the following alterations: a decrease in mRNA expression of NR2A receptor subunits both in hypothalamus and hippocampus; a decrease in mRNA expression of NR2B receptor in hypothalamus [37]. Reduced expression of both NR2A and NR2B receptors in hippocampus and hypothalamus are suggested to be involved in the underlying the mechanisms which leads to the onset of aggressive behavior.

**Fig. 3:**
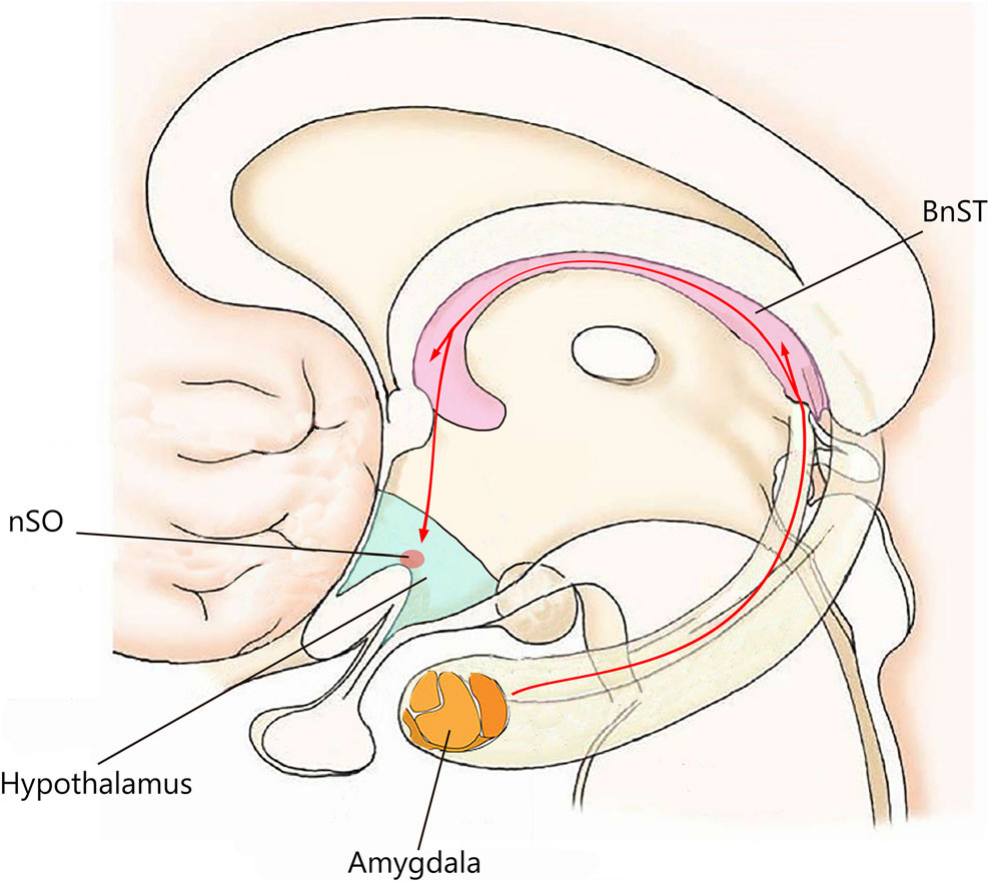
AAS-mediated neurotransmitter release and their action on latero-anterior hypothalamus (LAH): two different arrows are used to indicate inhibitory and excitatory action on LAH. These pathways are useful to clarify the AAS action on human behavior

Finally, other mediator involved in AAS-induced aggression seems to the dopamine receptors, whose role has been highlighted in a number of in vivo studies [4, 52]. Evidence shows that dopamine D_2_ receptor (D_2_R) located in the LAH directly modulate AAS-induced aggression in post-pubertal age. In addition, D_5_ receptors (D_5_R) have been indirectly linked to AAS-induced aggression. Likewise, dopamine released from LAH has been proven to influence GABAergic neurons via the inhibitory D_2_R; non-GABAergic neurons through the D_5_ receptor-mediated stimulation. Remarkably, D_5_R staining pattern observed in the LAH was similar to glutamatergic neurons pattern, since hypothalamus is densely populated with excitatory D_5_R and dopamine innervation to LAH after AAS exposure may facilitate aggression via the D_5_R [38, 53]. Moreover, dopamine release may influence glutamatergic neuron function across the neuraxis [54–56]. Moreover, Schwartzer et al. hypothesized that chronic AAS abuse may increase D_2_R activation in the LAH, and, thereby, inhibit GABA-mediated inhibition of AVP cells. Such D_2_R over stimulation finally results in the exacerbation of the aggressive behavior frequently observed in AAS abusers [4]. This hypothesis is supported by a number of studies suggesting that dopamine receptors particularly D2R and D5R activation triggers depolarization of neurons in the supraoptic nucleus of the hypothalamus [26]. Together with other neuromodulators, both dopaminergic pathways and activation of hypothalamic pathways forms the basis of AAS-related aggressiveness which is summarized in Fig. 3 [57].

Behind neurotransmitters, the redox status may also explain AAS-neurotoxicity. Indeed, Bueno et al. proved that different doses and periods of boldenone and stanazolol administration can change reactive oxygen species (ROS) levels in cerebral cortex and hippocampus [58]. Boldenone was also able to increase acetylcholinesterase (AChE) activity. This effect allowed suggesting that cholinergic system can be involved through the stimulation of presynaptic nicotinic acetylcholine receptors and consequently release of glutamate, GABA, dopamine, acetylcholine, norepinephrine, and serotonin. This mechanism could explain the increased aggressive-like behavior and territorial dominance [58].

## Conclusions

Considering the high prevalence and the increased frequency of AAS abuse documented worldwide and the detrimental effects of AAS on the behavior of users and their families is rapidly becoming a serious public health concern. The understanding of the precise underlying mechanisms and the pathophysiology of AAS-induced neuropsychiatric disorders is relatively recent and appears to be related to chronic AAS exposure. This subsequently leads to the development of anxiety and aggressive behavior. In order to prevent the onset of these symptoms, or to implement curative measures in chronic AAS abusers, a better understanding of the organic and functional processes involved in these psychiatric symptoms is required. Illicit AAS use and the related psychiatric symptoms may take a longer time to resolve, since other illicit substances such as alcohol and class A drugs are often also abused, especially in those with depression. In conclusion, AAS abuse is a global issue and requires more immediate primary and secondary prevention, which is only possible though more long-term research.
